# The Hypodermic Use of Corrosive Sublimate, in the Treatment of Syphilis

**Published:** 1872-03

**Authors:** Carl Proegler

**Affiliations:** Naperville, Illinois


ARTICLE I. — *The Hypodermic Use of Corrosive Sublimate, in the Treatment of Syphilis.* By CARL PROEGLER, M.D., Naperville, Illinois.

The aim of every kind of antisyphilitic treatment is to eliminate from the system all traces of the venereal poisoning. The treatment that will do this the most thoroughly, the most rapidly and the most agreeably to the patient, and is at the same time the most economic in the outlay of money and strength of the patient, ought to be the one that the physician should employ. The element of time should be largely considered in a country like ours especially, where every man's time is money. And if a treatment, embodying the points above named, can be used without necessitating any withdrawal from one's daily avocation, and requires no modification of diet or previous preparation of the system for receiving the medication, that course of treatment must commend itself at once.

To the sarsaparilla sweat-cure used some years in Germany, to the hunger-cure much in vogue at one time in France, the above meritorious points do not inhere. Besides, it is pretty well conceded by all syphilographers, that the only reliable antisyphilitic remedies are mercury and iodide of potassium. The latter remedy is not reliable for the severer forms of the disease. Relapses assuming the tertiary forms, viz.: periostitis, necrosis, rupia ulcerations, etc., are apt to follow the use of this medicine. It, too, is not potent enough to combat these forms of syphilis in the majority of cases. Even those who strongly incline to the anti-mercurialist's view, acknowledge that as an *ultimum refugium* mercury should be used, and that it does overcome the most unyielding forms of this disease. It is also believed that mercury is as thorough in all forms of syphilis as the most vaunted of treatment, since relapses after its use are as few, or fewer than after any other course of medication.

Since, then, mercury is an acknowledged remedy for syphilis, and in some cases the only sure one, the question now remains, *How and in what way shall it be used?*—by internal administration, by fumigation, by inunction, by introduction of mercurial suppositories, or by subcutaneous injection ?

Of these five ways, I think the latter is decidedly preferable. Although it is comparatively recent, yet the method has been so thoroughly tested that the physician who gives it a fair trial will greatly prefer it to the other forms of administration.

The internal use of mercury for the cure of syphilis is generally a protracted treatment, whether the drug is exhibited in the form of the iodides, chlorides, or in other forms.

For centuries, this drug has been blamed for producing salivation, stomatitis, dyscracia, and a general cachexia. By the subcutaneous employment of the drug, a much smaller quantity is necessary to be introduced into the system, and consequently the liability to ptyalism, and the bad effects of hydrargyrum are proportionably diminished and almost entirely avoided.

From statistics given by Prof. Lewin, Surgeon-in-Chief of the syphilitic wards of Charity Hospital, Berlin, where upwards of 2,000 patients have been treated by the subcutaneous method with the mercurial sublimate, we see that from 1½ to 3 grs. of the bichloride is all that was needed for the average of cases, including the most obstinate forms of tertiary syphilis.

So far as fumigation is concerned, it has no merits above the internal use for eradicating the disease, and it requires generally more time than most patients can well spare for each mercurial bath. Though strongly recommended by Langston it is very unpleasant to sensitive patients. Often this method is futile to overcome the disease.

The use of mercurial suppositories inserted into the rectum has been strongly recommended by Lebert, but the general verdict as to their efficacy is, that their results are unsatisfactory.

The hypodermic method, consisting of the use of a solution of the corrosive sublimate, was first successfully used and advocated by Prof. Lewin in the Berlin Charity Hospital, and also used by him in his private practice. He has somewhat modified the common hypodermic syringe which is inconvenient to use, and if used, an assistant is very necessary in order that the fold of skin may be held up and thus the fluid be delivered into the loose cellular tissue. If injected into the firm, unyielding subcutaneous tissue, much pain and even sloughing and abscesses may follow. Tiemann & Co., New York, are now making, by my suggestion, a syringe in accordance with Lewin's modification

The quantity used daily, depends somewhat on the gravity of the case. Ordinarily, doses from 1-12 to 1-4 grain are administered subcutaneously once each day. If the prodromes of salivation occur, a pause of three or four days may take place, when the treatment may be resumed. In urgent cases, sometimes 1-4 grain, twice daily, has been used by Prof. Lewin when the integrity of important organs have been attacked by the disease, *e. g.*, ulceration of the vocal chords or syphilitic iritis. When such large doses, however, are given, the toxic effect of the medicine may show itself to some degree.

Which solution to employ in any particular case, must rest upon the idiosyncrasies of the patient, his condition and the state of activity of the disease, etc. In ordinary cases it might be well to commence with the weaker and then adopt the stronger, if thought best. (I refer here, for particulars, to Prof. Lewin's book on syphilis, and its treatment by subcutaneous injections of sublimate, translated by Dr. Gale and myself, which will appear in a short time at Lindsay & Blakiston's, Philadelphia.)

When a patient exhibits a peculiarly sensitive diathesis, and whenever the sublimate injection is followed with pain, a small quantity of morphine may be added as a corrective. This will diminish the irritation and consequently the inflammation that might follow in highly sensitive persons, for *ubi irritatio, ibi fluxus.* With cases of this class we have added the morphine, or applied externally a chloroform liniment, and have never had any irritation or abscesses resulting.

So far as has come to my notice, but two *American* physicians have written anything respecting hypodermic sublimate injections—Dr. R. W. Taylor, Surgeon of the New York Dispensary (Med. Gazette, May 13, 1871, and cases), and Dr. F. R. Sturgis, of New York City (Journal of Syphilog. and Dermat., April, 1871.) The latter paper was only a review of the merits and demerits of the method, based upon the cases of others.

I requested Dr. Sturgis to give me his views, and he replied in a letter: "I do not doubt the ability to dissipate the symptoms by this treatment, but what advantages does it present over other methods, to wit, the inunction cure? Now, so far as I am able to judge, it has the disadvantage of pain, abscesses, and a more rapid induction of the toxical effects of corrosive sublimate."

The toxical effects can be avoided by a careful and judicious use of injections, if a suspension in the treatment takes place for two or more days when the same begin to manifest themselves.

The abscesses, Prof. Lewin thinks, are a result of an undextrous insertion of the fluid, he never having it occur in his private practice of 800 or 900 patients. Sometimes an abscess would form upon a hospital patient, only when a new assistant surgeon came to the hospital. But so soon as they acquired a little dexterity, such was never the case. Dr. Taylor, mentioned above, thinks abscesses may be wholly avoided, he having had only two patients suffering from them. Of course, there must be a trifling pain occasioned by the puncture, but it seems to me that any individual would, by far, rather endure this than to have the discomfort of a greasy, unclean apparel which exists with the inunction cure.

According to Bumstead's direction, the ointment should be smeared on at night, and that which remains on the skin must not be washed off in the morning, the same underwear being worn night and day. After the lapse of a week or ten days a bath may be taken, the linen changed, and the same repeated. Besides, those who have employed the subcutaneous method the most extensively, show, by a careful record of cases, that it is more rapid in its cure and more thorough, there being *thirty-five per cent. less of relapses* following it than any other antisyphilitic cure, whether mercurial or vegetable.

And then, again, the relapses which do follow, have a greater percentage of the lighter forms. The pain and discomfort felt from the pressure and distension occasioned by the presence of the liquid beneath the integuments, may be lessened and blunted by gently rubbing the parts, and thus causing the fluid to spread over a larger subcutaneous area.

And further, a suitable quantity of morphine added to the solution will obtund the local hyperæsthesia almost wholly. The advantages of the subcutaneous method and the frequent malignancy of syphilis should make every patient willingly undergo what little discomfort may unavoidably be connected with the method.

Dr. Taylor has treated fifty adult males and females subcutaneously for syphilis. His experience has detected striking merits as to the method, and certain disadvantages, he thinks, also. He is of the opinion, that the really secondary and even the later secondary rashes are quickly dissipated by this treatment and the rapidity with which the syphilitic neurosis disappears, and in syphilis of the nervous and osseous systems he thinks the hypodermic method is not so beneficial. In opposition to this opinion, we have the more extensive experience of Prof. Lewin, with his 2,000 cases; Prof. von Siegmund, of Vienna, (who has just completed his observations on 200 cases by a course of subcutaneous medication); Prof. Bamberger, of Vienna; Derblich, Bergson, etc.

Prof. Lewin in his work reports most excellent success in cerebral syphilis, even where paralysis has occurred; in necrosis of the bones from syphilis, where rupia and knotty syphiloids existed; in visceral syphilis; and, in fact, in every phase of the disease in its most advanced forms. The injections of sublimate vanquish the rheumatoid pains and the dolores osteocopei with remarkable quickness.

Prof. von Siegmund (Berl. Klin. Wochenschrift, N. 49, 1871), in commenting on his 200 cases recently treated with subcutaneous injections of sublimate, says, that there occurred but two abscesses in all, which resulted mainly from unskillful insertion of the liquid. He has seen no affection of the gums, no disturbances of the respiration or circulation, nor any other mercurial effects of any moment. He is so well pleased with this method of medication, that he states it to be his opinion, that all cases of syphilis will soon be treated subcutaneously with the injections of the corrosive sublimate.

I treated last year, in the German army, over 150 cases of syphilis by this method with perfect success, and also, conjointly with Dr. Gale, of Aurora, over 40 cases in private praxis, with complete success.

Probably when American practitioners have had as large an experience with the method as the above named high authorities, (Lewin and Siegmund), they will coincide with the latter gentleman as to its efficiency and practicability.

